# Fish Fin‐Derived Non‐Invasive Flexible Bioinspired Contact Lens for Continuous Ophthalmic Drug Delivery

**DOI:** 10.1002/advs.202412630

**Published:** 2024-12-16

**Authors:** Xu Li, Hui Li, Zihao Wang, Xianda Wang, Jinlong Zhang, Fengjiao Bin, Wei Chen, Hongyang Li, Dongmei Huo, Dengbao Xiao

**Affiliations:** ^1^ Institute of Advanced Structure Technology Beijing Institute of Technology Beijing 100081 China; ^2^ Beijing University of Technology Beijing 100124 China; ^3^ Beijing Friendship Hospital Capital Medical University Beijing 100050 China; ^4^ Shanghai East Hospital Shanghai 200120 China

**Keywords:** biomimetic microstructures, contact lens, drug delivery, flexible biomedical devices, non‐invasive

## Abstract

Efficient drug delivery is crucial for glaucoma patients. Flexible biomedical devices that enable sustained ocular drug delivery and can regulate the drug release rate according to physiological conditions are highly desirable for glaucoma treatments, addressing both low drug bioavailability and poor patient compliance from manual drug administration, and improving treatment outcomes. Inspired by the structure and reciprocating motion of fish dorsal fins, a drug‐eluting contact lens based on deformable microstructures for non‐invasive ocular surface drug delivery is developed. Liquid drugs are stored within the interstices of the deformable microstructural units, allowing for continuous drug release through diffusion upon contact with the ocular surface. Finite element analysis is utilized to study the intraocular drug transport dynamics of glaucoma and optimize the overall layout of the device. Microstructural units undergo deformation under loading, altering the interstitial spaces and modulating the drug release rate. This device can adaptively adjust its drug release rate based on changes in intraocular pressure (IOP) and can be proactively regulated in response to cyclic eye loads, accommodating elevated IOP caused by varying body postures and activities. As a flexible, non‐invasive, highly dynamic, and adaptive drug delivery platform, it holds significant potential for future biomedical applications.

## Graphical abstract

Inspired by the structure and reciprocating motion of fish dorsal fins, a drug‐eluting contact lens based on deformable microstructures for non‐invasive drug delivery is developed. The drug release rate can be adaptively adjusted to intraocular pressure changes and proactively regulated by cyclic ocular loads, enabling it to respond to elevated intraocular pressure from varying body postures and activities.

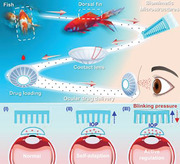

## 1 Introduction

The eye, as a crucial organ for the perception and transmission of visual information, plays an essential role,^[^ ^1^ ^]^ yet it is also threatened by numerous ocular diseases.^[^ ^2^ ^]^ Notably, glaucoma stands as the primary cause of irreversible blindness worldwide, with severe cases resulting in permanent vision loss.^[^ ^3^, ^4^, ^5^ ^]^ It is estimated that by 2040, glaucoma will affect 112 million people globally.^[^ ^6^ ^]^ The primary goal in treating glaucoma is to manage the intraocular pressure (IOP).^[^ ^7^ ^]^ Currently, the prevalent strategy for glaucoma treatment is the application of topical medications, predominantly eye drops, aimed at lowering IOP.^[^ ^8^, ^9^, ^10^ ^]^ However, conventional ocular drug delivery methods have significant limitations. Less than 5% of the administered medication is absorbed by the eye, due to dilution by tears, blinking, and physiological barriers such as the cornea.^[^ ^11^, ^12^, ^13^, ^14^ ^]^ Clinically, treatment often involves administering medication multiple times a day in a pulsed manner, requiring higher concentrations of the medication and frequent manual administration, inevitably leading to potential adverse effects on the human body.^[^ ^15^ ^]^ Patient adherence to glaucoma therapy is frequently compromised by memory lapses and hectic lifestyles, potentially leading to suboptimal treatment outcomes.^[^ ^16^ ^]^ Moreover, patients suffering from acute angle‐closure glaucoma, which is often accompanied by headache, nausea, and vomiting, may find it challenging to administer medication manually.^[^ ^17^ ^]^

Contact lenses that can continuously interact with tears are ideal drug release platforms.^[^ ^18^, ^19^, ^20^ ^]^ Drug‐eluting contact lenses can prolong the contact time between the drug and the ocular surface, thereby reducing the required dosage.^[^ ^21^ ^]^ In recent decades, wearable smart devices utilizing contact lenses for ocular drug delivery have been extensively researched to enhance drug bioavailability and improve treatment outcomes for glaucoma patients.^[^ ^22^, ^23^ ^]^ Many research strategies for drug delivery are based on the principle of passive diffusion, extending the drug's action time on the ocular surface through dissolution and diffusion processes.^[^ ^24^ ^]^ Self‐implantable micro‐needle drug reservoirs and biodegradable silicon nano‐needles embedded within contact lenses can achieve drug release in the eye for up to ≈50 days.^[^ ^25^, ^26^ ^]^ However, due to the prolonged degradation time required after absorption by the eyeball, it is difficult to interrupt the drug release, posing a risk of side effects from interactions with subsequent medications. Embedding microtubules within contact lenses as drug containers can provide continuous drug release and increase drug release through deformation caused by elevated IOP.^[^ ^27^ ^]^ Nonetheless, the drug release volume augmented by ocular deformation employing this structure is limited, similar to challenges faced by molecular imprinting technology,^[^ ^28^ ^]^ drug‐loaded hydrogels,^[^ ^29^, ^30^, ^31^ ^]^ and nanoparticles^[^ ^32^, ^33^ ^]^ in actively adjusting to varying physiological conditions of different patients. Triggering drug release through external stimuli as switches enables precise control over drug release timing, facilitating personalized drug application. Current research includes temperature,^[^ ^34^ ^]^ light,^[^ ^35^ ^]^ magnetic fields,^[^ ^36^ ^]^ electrical signals,^[^ ^37^, ^38^ ^]^ and pressure^[^ ^39^ ^]^ as triggers. Although drug‐loaded contact lenses employing active drug delivery technologies based on external stimuli can actively regulate drug release according to patient conditions, they often require additional devices or specific operations to trigger drug release, potentially compromising patient compliance, especially in long‐term treatments. Therefore, challenges may arise in clinical implementation due to these factors.

Glaucoma patients typically require long‐term treatment, and fluctuations in IOP are often significant, serving as a key indicator for diagnosing disease and measuring treatment effectiveness.^[^ ^40^ ^]^ Elevated IOP can cause irreversible damage to the retina and optic nerve, with higher IOP resulting in greater damage. Therefore, contact lenses used for ocular drug delivery should not only prolong the drug's action time on the eye and overcome the issue of poor patient compliance but should also increase drug release in response to high IOP, whether passively or actively, quickly releasing a larger amount of drug. When the IOP decreases, the released drug significantly reduces, helping avoid potential drug side effects.

In this work, inspired by the structure and reciprocating motion of fish dorsal fins, we develop a flexible biomimetic contact lens (FBCL) for non‐invasive ocular drug delivery. The FBCL features a biomechanically responsive multilevel drug release mechanism. Liquid drugs are stored within the interstices of the deformable microstructural units through capillary action and are continuously released through diffusion when in contact with the cornea, extending the drug's action time. Simultaneously, the deformable microstructures can respond to changes in IOP and externally applied ocular loads by the user, achieving both adaptive and active regulation of the drug release rate. Finite element analysis is utilized to study the intraocular drug transport dynamics of glaucoma and optimize the overall layout of the FBCL. Soft lithography combined with a 3D surface wrapping strategy is employed to fabricate the FBCL. Fluorescence diffusion experiments demonstrate that the FBCL can sustain continuous drug release for over 8 h a day with simple drug loading. Ex vivo experiments conducted on porcine eyes illustrate that the FBCL's drug release rate adapts to changes in IOP and can also be actively regulated based on cyclic ocular loads applied. In vivo animal experiments demonstrated that the FBCL effectively reduces the IOP in the high IOP model rabbit, indicating its favorable therapeutic efficacy under actual pathological conditions. This biomechanically driven dynamic drug‐release contact lens, which does not rely on external physical stimuli outside the human body, has the potential to become a new generation of ocular drug delivery devices.

## 2 Results and Discussion

### 2.1 Biomimetic Design and Working Mechanism of the FBCL

**Figure 1:**
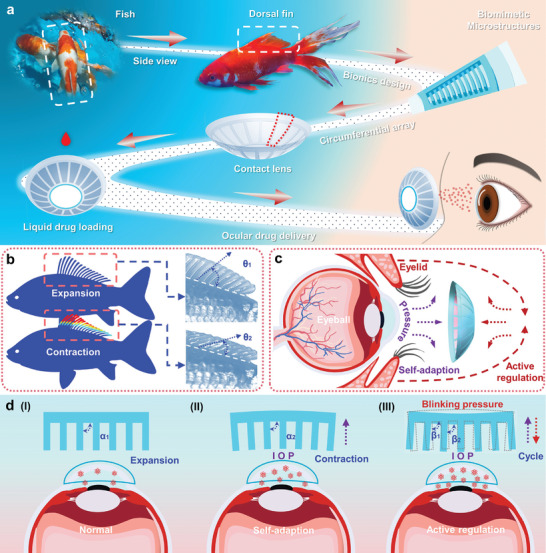
Design concept and working mechanism of the FBCL. a) Design principle diagram of the FBCL developed for ocular drug delivery. b) The different states of the dorsal fin structure and the corresponding angle changes between the fin and the fish body. c) The mechanism for adjusting the drug release rate of the FBCL. d) The principle of the multilevel drug release mechanism driven by deformable microstructures.

**Figure** **1**a illustrates the design principle diagram of the FBCL, developed for ocular drug delivery and inspired by the dorsal fin structure of aquatic fish in nature. The dorsal fin of fish species consists of several groups of skeletal arrangements spaced apart, extending the fin structure from a 2D plane to a 3D space resulting in a biomimetic microstructure, composed of several deformable microstructural units. The designed FBCL is obtained by arraying the biomimetic microstructures circumferentially and integrating them into the contact lens. Liquid drugs for the treatment of glaucoma can be stored in the interstices between the deformable units, achieving non‐invasive and sustained ocular drug delivery through diffusion upon contact with the cornea. Figure 1b illustrates two states of fish dorsal fin motion, including contraction and expansion of the fin. Such physical deformation mechanisms are widely observed in nature across various flora and fauna, including the wing structures of birds and the leaf movements of *Mimosa pudica*. Given the morphological consistency between the microstructure of the FBCL and the dorsal fin structure, the physical deformation mechanisms of dorsal fin contraction and expansion were analyzed to elucidate the corresponding mechanisms in the FBCL microstructure. In the expanded state, the angle θ_1_ formed between the dorsal fin and the fish body is larger, while in the contracted state, the angle θ_2_ formed between the dorsal fin and the fish body is smaller. These varying angles enhance the adaptability of fish in aquatic environments. The FBCL possesses a physical deformation mechanism analogous to that of the dorsal fin structure, enabling it to undergo corresponding physical deformations in response to external loads. This mechanism enables the regulation of drug release rates, allowing adaptation to the varying ocular environments of glaucoma patients. Figure 1c illustrates the regulation mechanism of drug release rates driven by biomechanical forces. The external loads acting on the FBCL primarily consist of pressure caused by IOP fluctuations and external pressure exerted during eyelid closure.

Figure 1d demonstrates the principle of a multilevel drug release mechanism driven by deformable microstructures. When the IOP is stable without drastic fluctuations, the microstructures are in an expanded state, and the angle α_1_ between the microstructures and the pole plate is relatively large. The drug stored in the interstices between the microstructures slowly interacts with the tear fluid through diffusion, resulting in a slower drug release rate, as illustrated in Figure 1d‐I. When the patient's IOP increases, the FBCL, which fits closely against the cornea, deforms accordingly. The microstructures are contracted, and the angle α_2_ between the microstructures and the pole plate decreases, which promotes the expulsion of the internal drug and accelerates the drug release. This state represents the adaptive regulation process of drug release by the FBCL, as depicted in Figure 1d‐II. In certain situations, when users feel discomfort due to increased IOP, they can apply cyclic pressure on the FBCL through the eyelid. Under the effect of cyclic pressure, the microstructures undergo cyclic deformation, analogous to the reciprocal movement of a fish fin structure. The angle between the microstructures and the pole plate cycles between β_1_ and β_2_, enhancing the liquid convection and thus increasing the drug release rate, as demonstrated in Figure 1d‐III.

### 2.2 Deformation Response and Intraocular Drug Transport Simulation

**Figure 2:**
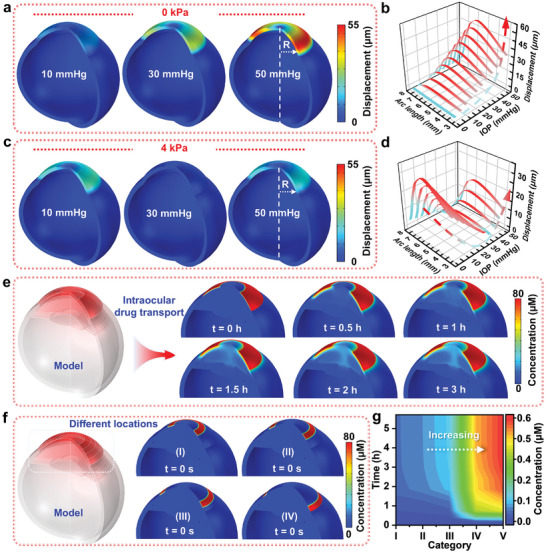
Deformation response and intraocular drug transport simulation. a) The deformation distribution of the model under different IOP conditions. b) The relationship between the deformation of FBCL's unilateral cross‐section (from the inner side to the corneal endpoint) and IOP. c) The deformation distribution of the model under different IOP conditions, including an external blinking load of 4 kPa. d) The relationship between the deformation of FBCL's unilateral cross‐section and IOP when subjected to an external blinking load of 4 kPa, with the displacement indicated solely by its magnitude. e) Finite element analysis of intraocular drug transport after applying the drug to the ocular surface utilizing the FBCL. f) The initial state of drug application at different locations on the cornea. g) Changes in drug concentration at the trabecular meshwork under the drug application conditions displayed in Figure 2f, where V represents the condition where the drug is applied to all four locations.

To observe the deformation response of the FBCL under fluctuations in IOP and cyclic ocular loads, a simplified ocular model consisting of the cornea, sclera, and the FBCL was established using COMSOL, as depicted in Figure S1 (Supporting Information). The FBCL is modeled as a 200 µm thick annular contact lens fitted to the corneal surface. Different uniform loads were applied to the model's inner surface to simulate varying IOP fluctuations, resulting in the deformation patterns illustrated in **Figure** **2**a, with deformation primarily concentrated on the cornea and the FBCL. The displacement on the FBCL was calculated utilizing COMSOL, revealing the deformation of the FBCL from the inner side to the end of the cornea under different IOP conditions, as depicted in Figure 2b. As the IOP increases, the deformation becomes more pronounced, indicating that the FBCL can effectively respond to IOP fluctuations, undergoing corresponding deformations to adaptively respond to these fluctuations. Humans naturally blink ≈10 000 times per day,^[^ ^41^ ^]^ accompanied by physiological behaviors such as eye movement and eyelid closure. However, these routine activities generally exert minimal pressure on the eyeball and are of short duration. Previous studies have reported that the average eyelid pressure during normal closure is ≈1.7 mmHg.^[^ ^42^ ^]^ Finite element simulations indicate that such mild ocular loads have negligible impact on the deformation of the FBCL, as demonstrated in Figure S2 (Supporting Information). However, the pressure from forceful blinking increases significantly,^[^ ^43^ ^]^ enough to exceed 50 mmHg. Finite element analysis here utilizes 4 kPa as an example,^[^ ^44^ ^]^ analyzing the effect of eyelid pressure on the corneal surface on the FBCL deformation under different IOP conditions. A uniform load of 4 kPa was applied to the pupil area of the cornea and the upper surface of the FBCL in the ocular model, resulting in the deformation distribution under different IOP conditions displayed in Figure 2c. As exhibited in Figure 2d, the pressure from IOP on the FBCL opposes the external pressure from the eyelid, resulting in a waveform‐like deformation of the FBCL. Under varying IOP conditions, the eyelid pressure on the corneal surface modifies the deformation of the FBCL, creating a displacement difference relative to the effects of light blinking, thus utilizing cyclic fluctuations to enhance the drug release rate of the FBCL.

As a sensitive organ of the human body, it is currently difficult to accurately determine the changes in drug concentration at a specific tissue inside the eyeball through experimental methods. Finite element simulation is an important method currently used to study the drug transport patterns inside the eyeball. Research utilizing finite element analysis has demonstrated that drug utilization from conventional local treatments, mainly involving eye drops, is very low but can be significantly enhanced through controlled drug release technologies.^[^ ^45^ ^]^ In order to further study the impact of applying drugs at different locations on the surface of the eyeball on the drug concentration at corresponding internal locations, and to aid in systematically designing the overall structure of the FBCL, an ocular model including the annular FBCL was established, as shown in Figure S3 (Supporting Information). The ocular model includes the main tissues inside the eyeball. The finite element analysis process considered the main physical processes within the eyeball, covering heat transfer from the external environment to the posterior segment of the eye, the secretion and discharge of aqueous humor, the natural convection of aqueous humor, and the transport of drugs within the eyeball after application through the FBCL. Figure S4 (Supporting Information), exhibits the boundary conditions of the ocular drug delivery model.

Ethacrynic acid is a drug recently considered to enhance the aqueous humor drainage capacity of the trabecular meshwork and is a potential glaucoma drug.^[^ ^46^ ^]^ Assuming that under the effect of the FBCL, the drug concentration on the external surface of the cornea remains constant in the short term. Figure 2e demonstrates the drug transport situation inside the eyeball when the drug concentration on the external surface of the cornea is 80 µm. After the drug is applied through the FBCL, most of the drug enters the anterior chamber through diffusion, and a small portion of the drug crosses the sclera to enter the trabecular meshwork and is expelled. Due to the presence of physiological barriers, the drug concentration inside the eye is much lower than the drug concentration applied on the surface of the cornea. Finite element simulation was used to study the impact of drug concentrations at four different locations from the inside to the outside of the cornea on the drug concentration at the trabecular meshwork inside the eyeball. Figure 2f exhibits the initial state of applying drugs at different locations on the cornea. Figure S5 (Supporting Information), shows the drug distribution inside the eye 2 h after applying drugs at different locations on the cornea. Comparing the changes in drug concentration at the trabecular meshwork after applying drugs at each of the four locations and after applying drugs at all four locations, as illustrated in Figure 2g, the drug concentration at the trabecular meshwork first increased and then reached a peak. After comparison, it was found that the closer the drug application location is to the trabecular meshwork, the better the effect. However, the effect of applying the drug alone is not as good as that of applying the drug overall. This principle can be used to optimize the overall structure of the FBCL, allowing it to achieve sustained drug release while maintaining a high drug concentration in the trabecular meshwork inside the eye.

### 2.3 System Design and Fabrication of the FBCL

**Figure 3:**
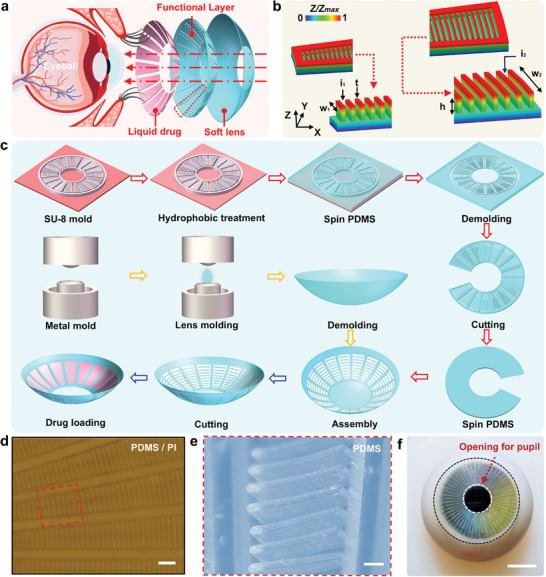
System design and fabrication of the FBCL. a) Schematic of the overall 3D structure of the FBCL. b) Distribution of microstructure dimensions. c) Fabrication process of the FBCL. d) Optical image of the microstructure layer. Scale bar: 200 µm. e) Partial 3D view of the microstructure. Scale bar: 100 µm. f) Optical image of the FBCL on an artificial eyeball. Scale bar: 5 mm.

The FBCL consists of two layers of polydimethylsiloxane (PDMS), one layer is a flexible contact lens base that maintains the shape of the FBCL, and the other layer is a functional layer with deformable biomimetic microstructures for loading liquid drugs. PDMS is known for its flexibility and biocompatibility, and can easily be modified into a hydrophilic material, often utilized as the material for contact lenses.^[^ ^38^, ^47^ ^]^ Due to the presence of microstructures which may affect vision, the functional layer of the FBCL has a hollow area larger than the pupil at its center to ensure light can enter the pupil, as illustrated in **Figure** **3**a. The layout of the microstructure layer adopts a spider‐web‐like arrangement, as demonstrated in Figure S6 (Supporting Information), which on one hand increases the drug loading capacity and improves the spatial utilization of the FBCL, and on the other hand, the spider‐web‐like structural layout compared to constant‐width radial layout achieves higher drug concentrations near the trabecular meshwork, which is beneficial for increasing the drug concentration at the trabecular meshwork. Figure S7 (Supporting Information), displays the detailed planar microstructure layout design parameters. Figure 3b describes the 3D structure of a single microstructure in the circumferential array of the microstructure layer, with the microstructure unit's height (h) being 100 µm and thickness (t) being 25 µm. The interstice between microstructures determines the amount of drug loaded. Too small an interstice between microstructure units leads to a lower amount of drug loaded, while too large an interstice may cause the drug to be released too quickly, losing the effect of prolonging the duration of drug action. The width (w_2_) of the microstructures at the outermost position is greater than the width (w_1_) of the microstructures at the innermost position, thus allowing for larger interstices near the edges. According to the drug transport patterns within the eyeball, the interstice (i_2_) between microstructure units in the area shown in Figure 2f‐IV is set at 45 µm, while the interstice (i_1_) between microstructure units in the remaining area is 35 µm. This not only extends the duration of the drug's action but also further increases the average drug concentration in the area near the trabecular meshwork.

Figure 3c illustrates the process of fabricating the FBCL utilizing soft lithography techniques and a 3D surface wrapping strategy. The fabrication of FBCL involves creating the bionic microstructure layer and the flexible contact lens base. Standard photolithography was utilized to create 100 µm thick SU‐8 microstructure patterns on a silicon substrate, resulting in an SU‐8 mold. The SU‐8 mold was silanized to ensure smooth demolding of the PDMS film. PDMS and curing agent were mixed in a 10:1 ratio, uniformly spin‐coated on the silicon mold, and transferred to a drying oven for curing. The PDMS film with biomimetic microstructures was carefully peeled off and notched to facilitate assembly with the flexible contact lens base. The PDMS contact lens is molded by pouring the prepared PDMS mixture into the lens mold, combining the two parts of the mold, and demolding after curing to obtain the lens base, as illustrated in Figure S8 (Supporting Information). Finally, a layer of PDMS was spin‐coated on the back of the cut microstructure layer, and it was assembled with the prepared contact lens base through a secondary curing process. After curing, the contact lens was trimmed to remove the central field of vision area, and after loading with drugs, it could be used for drug delivery on the surface of the eyeball. Figure 3d displays the optical image of the microstructure layer placed on the PI film, while Figure 3e shows the partial 3D image at the corresponding position in Figure 3d,f displays the optical image of the FBCL worn on an artificial eyeball, demonstrating that the annular design allows light to pass through the central area to the pupil.

### 2.4 Drug Release Experiment of the FBCL

**Figure 4:**
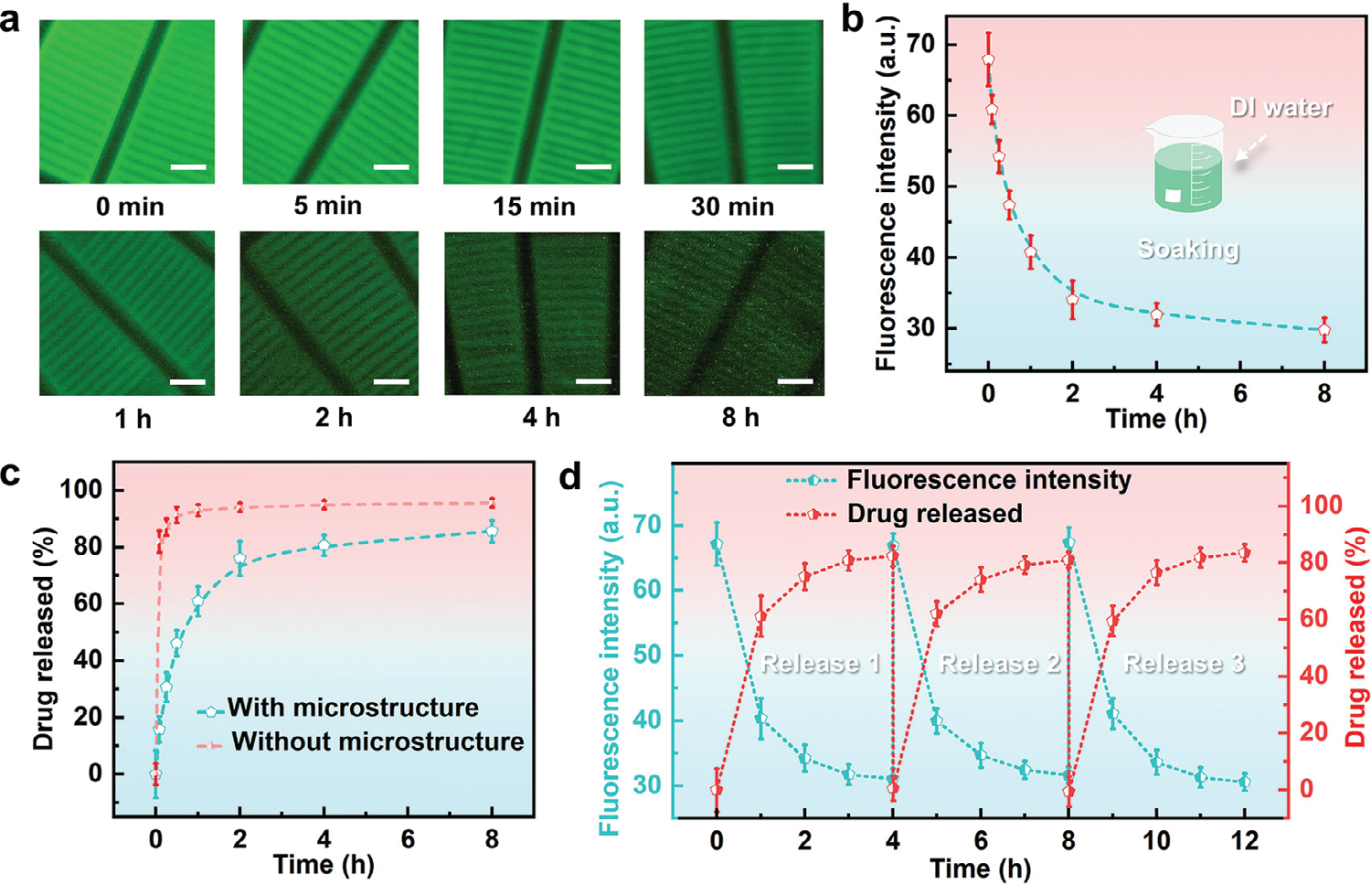
Drug release experiment of the FBCL. a) Representative fluorescence images of the drug‐loaded microstructure over time. Scale bar: 200 µm. b) Changes in average fluorescence intensity (*n* = 6). c) Comparison of drug release rates between the FBCL and PDMS contact lens without microstructures (*n* = 6). d) Cycle testing of the FBCL (*n* = 6).

Patients with glaucoma often require long‐term treatment. To validate the capability of the FBCL for sustained ocular surface drug delivery, meeting the continuous treatment needs of glaucoma patients, the drug release function of the FBCL was tested through fluorescence experiments. The primary treatment for glaucoma currently involves eye drops. A mixture of fluorescein sodium salt and deionized water was used to simulate small molecular soluble drugs. To quantify drug release, solutions of fluorescein sodium at different concentrations were loaded onto the microstructure layer, and corresponding fluorescence images were captured. The average fluorescence intensity at various concentrations was calculated utilizing ImageJ, establishing a relationship between average fluorescence intensity and drug concentration, as illustrated in Figure S9 (Supporting Information). The fluorescence intensity calculation only considered the upper surface of the microstructure units and the interstices, excluding the partitions in the middle of the annular array. The average fluorescence intensity increased with the drug concentration, and based on this correlation, the drug release from the microstructure layer could be quantified. The microstructure layer in the FBCL was loaded with a 0.1% w/v solution of sodium fluorescein, then placed on an artificial eyeball and immersed in 8 mL of deionized water, ensuring that the drug concentration in the deionized water remained lower than that in the microstructure layer, thereby guaranteeing sustained drug release. At regular intervals, the FBCL was taken out and the corresponding fluorescence images were captured. Representative fluorescence images showing the change in fluorescence intensity of the drug‐loaded microstructure layer over time are illustrated in **Figure** **4**a.

From the fluorescence images of the local microstructures, it can be observed that the drug mainly resides in the interstices between microstructure units, and the fluorescence intensity gradually decreases over time. The average fluorescence intensity of the drug‐loaded microstructure at different times was calculated using ImageJ, as depicted in Figure 4b. The fluorescence intensity decreases most significantly within the first 2 h, then the rate of decrease slows down. Figure 4c compares the drug release rate of the FBCL with a PDMS contact lens without microstructures under the same conditions. The PDMS contact lens without microstructures quickly released over 90% of the drug. The drug‐loaded microstructure layer in the FBCL effectively slows the release rate. Although nearly 80% of the drug is released within the first 2 h, the overall drug release time can last up to 8 h, sufficient for daily use. The drug‐loaded microstructure layer in the FBCL does not involve complex drug‐loading procedures and can be achieved through simple operations, thus realizing long‐term ocular drug delivery. Figure 4d illustrates the drug release experiments of the FBCL under three cycles, demonstrating good stability and repeatability. Short‐term drug loading significantly increases the drug concentration in the microstructure layer, thus better facilitating long‐term treatment of ocular diseases.

### 2.5 Ex Vivo Porcine Eye Experiment of the FBCL

**Figure 5:**
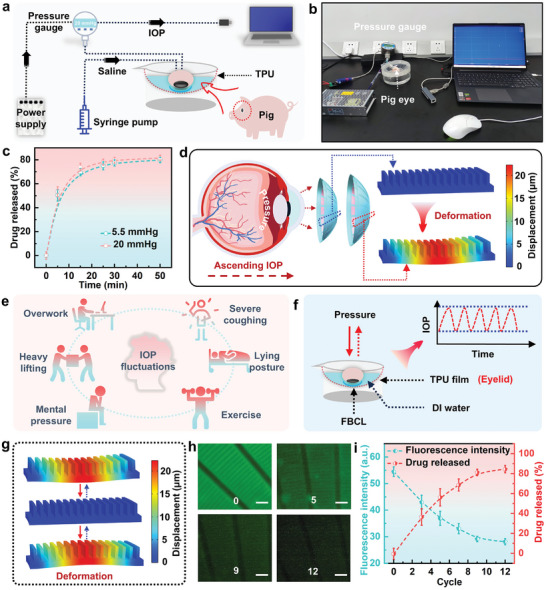
Ex vivo porcine eye experiment of the FBCL. a) Schematic of the experiment based on the ex vivo porcine eye. b) Actual image of the experimental platform. c) IOP‐driven adaptive drug release experiment (*n* = 6). d) Schematic of the FBCL shape changes and internal microstructure adaptive deformation simulation when IOP increases. e) Factors inducing high IOP in daily life. f) Schematic of the experiment simulating cyclic ocular loads. g) Simulation of microstructure deformation under cyclic ocular loads. h) Representative images of microstructures in the FBCL at different cycle counts. Scale bar: 200 µm. i) Relationship between drug release amount and number of cycles (*n* = 6).

The size and mechanical properties of porcine corneas are relatively similar to those of human eyes and are widely used in contact lens experiments.^[^ ^48^, ^49^ ^]^ An experimental platform based on the ex vivo porcine eye was established to verify the multilevel drug release mechanism of the FBCL. Figure 4a,b respectively exhibit the experimental principle and the actual image of the constructed experimental platform. Two syringes were inserted into the ex vivo porcine eye to adjust and record the internal IOP in real time, simulating changes in the eye's internal IOP. A curved depression was created on a petri dish utilizing a layer of TPU film (50 µm), and during the experiment, the microstructures were loaded with a 0.07% v/w solution of sodium fluorescein. The FBCL was worn on the ex vivo porcine eye and placed upside down on the TPU film, and ≈15 mL of deionized water was poured to soak the porcine eye while preventing it from floating. A syringe pump was utilized to adjust the IOP of the ex vivo porcine eye. Periodically, the FBCL was removed, fluorescence images of the microstructures were captured, and the drug release rate of the FBCL under different IOP conditions was measured. Due to the difficulty in maintaining a high IOP in the ex vivo porcine eye for extended periods, which can lead to fluid leakage, the drug release rates of the FBCL at 5.5 mmHg and 20 mmHg were measured, as illustrated in **Figure** **5**c. At 20 mmHg, the drug release rate of the FBCL was higher than at 5.5 mmHg, demonstrating the FBCL's adaptive drug release function. As the IOP increases, the FBCL undergoes deformation due to compression by the eyeball, changing the interstices between the microstructures, as displayed in Figure 5d, thereby promoting drug expulsion. The boundary conditions for the finite element simulation of the deformation mechanism of the microstructure under external loads are presented in Figure S10 (Supporting Information).

In glaucoma patients, the IOP is susceptible to dramatic fluctuations due to factors such as body posture and physical activity, potentially causing irreversible damage to the optic nerve. Many daily activities can trigger high intraocular pressure, as illustrated in Figure 5e. Although adaptive drug release can respond to changes in IOP, this process is slow and weak, necessitating a further acceleration of drug release rate under high IOP to alleviate its detrimental effects on the optic nerve. Figure 5f illustrates the experimental schematic diagram of simulating cyclic ocular load using the ex vivo pig eye. By applying cyclic loads to simulate the process of a user blinking forcefully, the drug release rate of the FBCL's responsiveness to user‐controlled actions (forceful blinking) is tested. During the application of load, the IOP of the porcine eye undergoes cyclic fluctuations, continually increasing during loading and slowly decreasing upon load removal. The IOP of the porcine eye measured in real‐time with the pressure gauge serves as the control standard for load application, regulating the IOP to fluctuate from 6 ± 1to 20 ± 2 mmHg with each cycle lasting ≈0.5 min (Movie S1, Supporting Information). Based on predetermined cycle numbers, the FBCL is periodically removed and its fluorescence images are captured to quantify the amount of drug released. COMSOL simulation is employed to model the structural changes of microstructures under external loading. During this process, microstructures undergo a transition between expansion and contraction states, which would accelerate convection between the drug and solution, as illustrated in Figure 5g,h presents representative fluorescent images of microstructures in the FBCL after undergoing different numbers of cycles, with the fluorescence intensity of microstructures decreasing with increasing cycle numbers. The relationship between drug release amount and number of cycles is illustrated in Figure 5i, where cyclic loading applied to porcine eyes prompts an increase in the amount of drug released by the FBCL, demonstrating its satisfactory responsiveness.

### 2.6 In Vivo Animal Experiment of the FBCL

**Figure 6:**
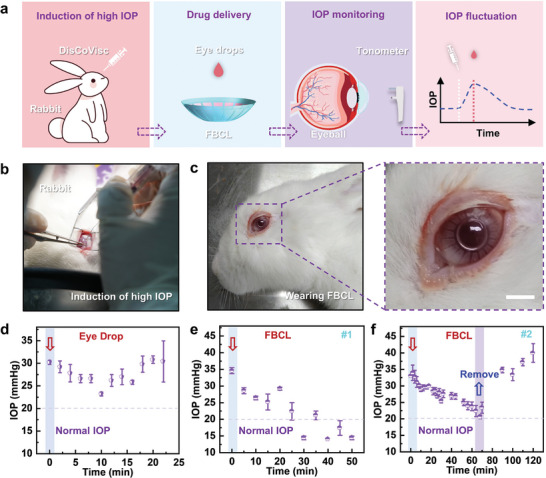
In vivo animal experiment of the FBCL. a) Schematic diagram of the in vivo animal experiment. b) Photograph of the experiment inducing high IOP. c) Physical image of the experimental rabbit wearing the FBCL, with an enlarged view showing a close‐up of the FBCL. Scale bar: 6 mm. d) Changes in IOP in the experimental rabbit with high IOP after administration of eye drops (*n* = 5). Red arrow indicates the time of eye drop application. e) Changes in IOP in experimental rabbit #1 with high IOP during ocular drug delivery via the FBCL (*n* = 5). Red arrow indicates the time of the FBCL placement. f) Changes in IOP in experimental rabbit #2 with high IOP during ocular drug delivery via the FBCL (*n* = 5). Red arrow marks the placement time of the FBCL, while the blue arrow denotes its removal.

Elevated IOP is one of the main characteristics of glaucoma. Conducting in vivo animal experiments can assess the efficacy of FBCL in reducing IOP under actual pathological conditions. The schematic diagram of the in vivo animal experiment is shown in **Figure** **6**a. By inducing high IOP in rabbit eyes, an animal model with high IOP similar to human glaucoma is established. The FBCL is loaded with eye drops and placed on rabbit eyes in a high IOP state for drug therapy. Considering that the experimental rabbits do not blink to adjust to high IOP like humans, the variation in drug release rate from the FBCL was not considered during the experiment. Instead, a tonometer was used to regularly measure the IOP of the rabbit eyes, capturing the trend of IOP changes over time to analyze and evaluate the efficacy of FBCL in lowering IOP under actual pathological conditions. Figure 6b shows the surgical process of inducing high IOP in the rabbit eye. The photographs demonstrate that there was no leakage of intraocular fluid during the procedure. Approximately 0.5 mL of viscoelastic agent was injected into the rabbit eye to induce a high IOP state. The injection of the viscoelastic agent increases the intraocular volume and pressure, thereby raising the IOP. The physical image of the experimental rabbit wearing the FBCL, as well as an enlarged image of the FBCL, are shown in Figure 6c. Due to the differences in the corneas of rabbit eyes, the FBCL worn on rabbit eyes was redesigned. A curved mold was fabricated using 3D printing to quickly fabricate an FBCL that fits the rabbit eyes, as illustrated in Figure S11 (Supporting Information). Additionally, the dimensional parameters of the microstructure were adjusted to increase the interstices, thereby enhancing the drug release rate and reducing the time the experimental rabbits wore the FBCL, to avoid complications caused by prolonged wear that could interfere with the experimental results. The FBCL was pre‐soaked in the eye drops and was removed for the experiment 5 min prior to the experiment.

Following surgical induction, the IOP in the experimental rabbit increased to a pathological level of high IOP and gradually stabilized, as shown in Figure S12 (Supporting Information). Treatments included conventional eye drop administration and drug delivery via the FBCL. Figure 6d presents the result of treatment with traditional eye drops, where each data point represents the mean of five measurements, and the error bars represent standard deviations. Brinzolamide and Timolol Maleate Eye Drops (10 mg mL^−1^ Brinzolamide and 5 mg mL^−1^ Timolol Maleate, 0.05 mL^−1^) were directly instilled into the eye of a high‐IOP rabbit. Initially, IOP decreased, but the effect rapidly diminished over time, failing to reduce IOP to normal levels and subsequently rebounding to the high IOP state. Figure 6e illustrates the IOP changes in high‐IOP rabbit #1 after the FBCL placement. Within the first 30 min, IOP gradually decreased from the initial high level to normal values and then fluctuated within the normal range, demonstrating the efficacy of the FBCL. Figure 6f shows the IOP changes in high‐IOP rabbit #2, monitored every 2 min from 0 min onward, with five measurements recorded at each time point. In contrast to rabbit #1, the FBCL in experimental rabbit #2 was removed once the IOP was restored to normal levels. Subsequently, the IOP was elevated to pathological levels again, effectively eliminating the potential influence of intrinsic factors associated with the rabbit model. Therefore, in vivo animal experiments demonstrate that drug delivery via the FBCL exhibits significant efficacy under pathological conditions by effectively reducing high IOP and maintaining it within the normal range, outperforming conventional treatment methods.

## 3 Conclusion

In summary, this work presents a flexible drug‐loaded contact lens inspired by fish fin structures for non‐invasive ocular drug delivery. The contact lens incorporates a multilevel drug release mechanism based on biomechanical response. Liquid drugs, stored in the interstices of deformable microstructures, are released sustainably through diffusion upon contact with the cornea. Furthermore, these microstructures respond to changes in IOP and external ocular loads such as blinking, allowing for both adaptive and user‐driven adjustments in drug release rates. Finite element simulation analyzes intraocular drug transport patterns in glaucoma patients, guiding the optimization of the overall layout of the FBCL. The contact lens was fabricated through soft lithography combined with a 3D surface coating strategy. Fluorescent diffusion experiments demonstrate that the contact lens can sustain drug release for 8 h a day and can be reused multiple times. In vitro experiments on excised porcine eyes confirm that the drug release rate of the contact lens adapts to changes in IOP and can be actively adjusted based on cyclic ocular loads. The in vivo animal experiment demonstrated that the FBCL effectively reduces the IOP in the high IOP model rabbit, indicating its favorable therapeutic efficacy under actual pathological conditions. This dynamic drug‐releasing contact lens, driven solely by biomechanical forces without relying on external physical stimuli, holds tremendous potential for future applications (Table S1, Supporting Information). As the fish fin‐inspired design increases lens thickness, future advancements in micro‐nano manufacturing and structural optimization could address this challenge. The current structure exhibits limited sensitivity to IOP fluctuations, and future efforts could focus on improving this sensitivity. Moreover, considering the interindividual variability in drug efficacy, personalized customization holds significant potential to further enhance therapeutic outcomes, paving the way for more precise and effective treatments.

## 4 Experimental Section

### Fabrication of the FBCL

The SU‐8 mold was manufactured using microfluidic processing technology by Yuan Dian Technology Co., Ltd., Guangzhou, China, followed by a hydrophobic treatment. The PDMS solution (Sylgard 184, Dow Corning, USA) and curing agent were prepared in a 10:1 ratio and thoroughly mixed using a magnetic stirrer (M‐SCL‐T, Hangzhou Qiwei Instrument Factory, China) at 1600 rpm for 18 min. Subsequently, the air bubbles in the well‐mixed PDMS solution were removed in a vacuum environment provided by a vacuum drying oven (DZF‐6096, Shanghai Yiheng Scientific Instruments Co., Ltd., China) at 10 Pa for 25 min. The PDMS solution was then spin‐coated onto the surface of the SU‐8 mold using a desktop coating machine (KW‐4B‐I, Beijing Sedekais Electronics Co., Ltd., China) and transferred to the vacuum drying oven for curing. Initially, the solution was cured at 60 °C for 30 min, followed by 80 °C for 1.5 h. After curing, the mold was carefully removed, and the PDMS microstructures were peeled off. Next, the bubble‐free PDMS solution was poured into a stainless steel mold and cured again in the vacuum‐drying oven to fabricate the PDMS contact lens. The PDMS contact lens and microstructure layer were cut into a ring shape with notches, and a layer of PDMS solution was spin‐coated onto the back of the microstructure layer. These were assembled on a concave mold and transferred back to the vacuum oven for a second curing process. Finally, the PDMS contact lens was removed and cut into a ring shape.

### Drug Model and Fluorescence Calibration

Fluorescein sodium salt (Tianjin Dengfeng Chemical Reagent Factory, China) was mixed with deionized water to simulate small molecule water‐soluble drugs. Different concentrations of fluorescein sodium solutions were prepared and loaded onto the microstructures. An optical microscope (AO‐4K32C, Shenzhen Oustar Micro‐Optical Co., Ltd., China) was equipped with a 500–550 nm green light high‐transmission filter (2.45 mm, Shenzhen Infrared Laser Technology Co., Ltd., China) and a four‐light source flashlight (A100, Yiwu Zojun E‐commerce Co., Ltd., China), serving respectively as the emission filter and fluorescence excitation light source, thus converting the optical microscope into a fluorescence microscope,^[^ ^50^ ^]^ as indicated in Figure S13 (Supporting Information). The entire experiment was conducted in a dark laboratory environment to prevent sunlight of corresponding wavelengths from entering the microscope. The modified fluorescence microscope was used to obtain fluorescence microscopy images of the microstructures loaded with various concentrations of fluorescein sodium solution. Using ImageJ, the average fluorescence intensity within the microstructure units and the interstices was calculated to establish the relationship between fluorescence intensity and drug content. Since ordinary PDMS films do not contain microstructures, the fluorescence intensity of PDMS films under different drug concentrations was calibrated to facilitate subsequent comparisons.

### Drug Release Experiment of the FBCL

The temperature and humidity in the laboratory environment were measured using a thermo‐hygrometer (MBS‐323, MBOOS, Germany) and recorded as 20 ± 1 °C and 50 ± 5% respectively. PDMS contact lens of the same size as the FBCL were cut for comparison. Both were loaded with a 0.1% w/v solution of sodium fluorescein and immersed in 8 mL of deionized water along with an artificial eyeball. At regular intervals, they were removed to capture fluorescence images. The average fluorescence intensity was calculated using ImageJ software, and the amount of released drug was determined based on the established relationship. The drug release results from the PDMS contact lens were obtained based on the calibration performed separately on the PDMS film.

### Experiment Simulating the FBCL Response to IOP Fluctuations

Fresh ex‐vivo porcine eyes were sourced from the Beijing Hengkangjian Trade Center. To prevent dehydration‐induced shape changes, the porcine eyes were soaked in saline prior to the FBCL experiments. IOP adjustments and real‐time monitoring were achieved by inserting two intravenous catheter needles. A 24‐gauge intravenous catheter was connected to a high‐precision pressure gauge (YBS80A, YBPCM, China) to measure IOP changes, while a 26‐gauge catheter was connected to an infusion pump to control IOP by adjusting saline infusion. A 50 µm TPU film was used to create a concave pit in a Petri dish, where the FBCL was fitted onto the ex‐vivo porcine eye and inverted inside the dish. Needles were inserted, and ≈15 mL of deionized water was added to the TPU film to soak the FBCL and prevent the porcine eye from floating. The infusion pump adjusted the IOP, and the FBCL was periodically removed to capture fluorescent images of its microstructure and measure drug release rates at 5.5 mmHg and 20 mmHg.

### Experiment Simulating the FBCL Response to Ocular Loads

Downward loads were applied to ex‐vivo porcine eyes to simulate ocular loading, compressing the FBCL between the porcine eyeball and a TPU film. To prevent potential fluid leakage from excessively high IOP, which could affect the experimental results, the initial IOP of the porcine eyes was set to a lower level at the beginning of the experiment. By applying loads, the IOP of the porcine eyes was cycled between 6 ± 1 and 20 ± 2 mmHg, with each cycle lasting ≈0.5 min. The FBCL was periodically removed to capture fluorescent images of its microstructure, quantifying the amount of drug released.

### In Vivo Animal Experiment of the FBCL

Four male New Zealand white rabbits (2.5–3 kg, sourced from Changyang Xishan Breeding Farm and Rich Experimental Animal Breeding Center, Beijing) were used for intraocular drug release experiments. The rabbits underwent one week of adaptive feeding with a 12‐h light/dark cycle prior to the experiment. All in vivo animal experiments were reviewed, approved, and supervised by the Animal Ethics Committee of Beijing Jinglai Huake Biotechnology Co., Ltd. (JLHK‐20240715‐01) and the Animal Ethics Committee of Kangtai Medical Laboratory Service Hebei Co., Ltd. (MDL2024‐11‐09‐01), in accordance with ethical regulations. After the adaptive feeding period, the rabbits were anesthetized and immobilized. Povidone‐iodine was applied for local disinfection, and baseline IOP was measured using a tonometer (SW‐500, Tianjin Suowei Electronic Technology Co.,Ltd., China). A three‐plane incision was made at the temporal limbus using a 1.8 mm ophthalmic knife, followed by the injection of ≈0.5 mL of viscoelastic agent (DisCoVisc, Alcon, USA) into the anterior chamber to elevate IOP. IOP was monitored continuously, and the experiment began once IOP increased and stabilized. One New Zealand rabbit was left untreated to monitor changes in IOP following the induction of high IOP surgery, while another rabbit was treated with traditional eye drop administration. The remaining two rabbits were subjected to drug delivery via the FBCL. Before the experiment, the FBCL was soaked in Brinzolamide and Timolol Maleate Eye Drops (Azarga, S.A. Alcon‐Couvreur N.V., Belgium) and then fitted onto the two rabbits. The IOP of each rabbit was measured and recorded at regular intervals using a tonometer.

### Finite Element Simulation Analysis

Finite element analysis was conducted using the commercial software COMSOL (version 6.0, COMSOL, Stockholm, Sweden) to investigate the deformation of the FBCL under different IOP conditions and ocular loading, the deformation of the local microstructure under loading, and the transport of the drug within the eye.

### Statistical Analysis

The quantitative data presented in the figures of this paper are expressed as mean ± standard deviation, with the standard deviation serving as the representation for the error bars. The statistical analysis and data visualization were conducted using Microsoft Excel 2019 and Origin 2022 software.

## Conflict of Interest

The authors declare no conflict of interest.

## Author Contributions

X.L. and H.L. contributed equally to this work. D.X. and X.L. conceived the concept. X.L. designed the work, conducted the finite element simulation, designed the experiments, and wrote the manuscript. X.L., H.L., and Z.W. built the experimental platform and fabricated the devices. X.L., H.L., W.C., J.Z., X.W., H.Y. L., D.H., and F.B. performed the experiments. X.L., H.L., W.C., X.W., and D.X. analyzed the data. D.X. supervised the project. All authors reviewed and advised on the manuscript.

## Supporting information



Supporting Information

Supplemental Movie 1

## Data Availability

The data that support the findings of this study are available from the corresponding author upon reasonable request.
