# Supplementary material for: Fish Fin‐Derived Non‐Invasive Flexible Bioinspired Contact Lens for Continuous Ophthalmic Drug Delivery
Source: Adv Sci (Weinh). 2024 Dec 16;12(6):2412630. doi: 10.1002/advs.202412630 (PMC11809385; doi:10.1002/advs.202412630)
Supplement: Supplementary file 1 — Supporting Information [file ADVS-12-2412630-s001.pdf]

ADVANCED SCIENCE
Open Access

Supporting Information

for *Adv. Sci.*, DOI 10.1002/advs.202412630

Fish Fin-Derived Non-Invasive Flexible Bioinspired Contact Lens for Continuous Ophthalmic Drug Delivery

Xu Li, Hui Li, Zihao Wang, Xianda Wang, Jinlong Zhang, Fengjiao Bin, Wei Chen*, Hongyang Li, Dongmei Huo and Dengbao Xiao**

Supporting Information

Fish Fin-Derived Non-Invasive Flexible Bioinspired Contact Lens for Continuous Ophthalmic Drug Delivery

Xu Li[†,], Hui Li[†], Zihao Wang, Xianda Wang, Jinlong Zhang, Fengjiao Bin, Wei Chen*, Hongyang Li, Dongmei Huo, Dengbao Xiao**

X. Li, H. Li, Z. Wang, X. Wang, J. Zhang, F. Bin, D. Xiao
Institute of Advanced Structure Technology
Beijing Institute of Technology
Beijing 100081, China
E-mail: lixu96069@163.com (X. Li); xiaodengbao@bit.edu.cn (D. Xiao)

W. Chen
Beijing University of Technology
Beijing 100124, China
E-mail: Chenwei@bjut.edu.cn (W. Chen)

H.Y. Li
Beijing Friendship Hospital
Capital Medical University
Beijing 100050, China

D. Huo
Shanghai East Hospital
Shanghai 200120, China

X.L. and H.L. contributed equally to this work.

The file includes:

Figure S1. Ocular model incorporating the cornea, sclera, and the FBCL, constructed using COMSOL.
Figure S2. Deformation simulation of the FBCL under mild ocular load.
Figure S3. Intraocular drug transport model with the annular FBCL.
Figure S4. Boundary conditions of the intraocular drug transport model.
Figure S5. Drug concentration distribution within the ocular region after application at different locations on the cornea.
Figure S6. Layout choices in the design process of the FBCL.
Figure S7. Design parameters of microstructural units in the planar microstructure layout.
Figure S8. Manufacturing of the contact lens base and its assembly with the PDMS microstructure layer.
Figure S9. Drug loading image and fluorescence calibration.
Figure S10. Boundary conditions and application methods of different boundary loads in the microstructure deformation simulation.
Figure S11. Physical image of the curved mold prepared using 3D printing.
Figure S12. IOP variations in the rabbit eye after high-IOP induction surgery.
Figure S13. Modified fluorescence microscope experimental platform.
Table S1. Comparison between this work and previous related drug delivery devices
Movie S1. Demonstration of cyclic fluctuations in intraocular pressure of pig eyes under external load.

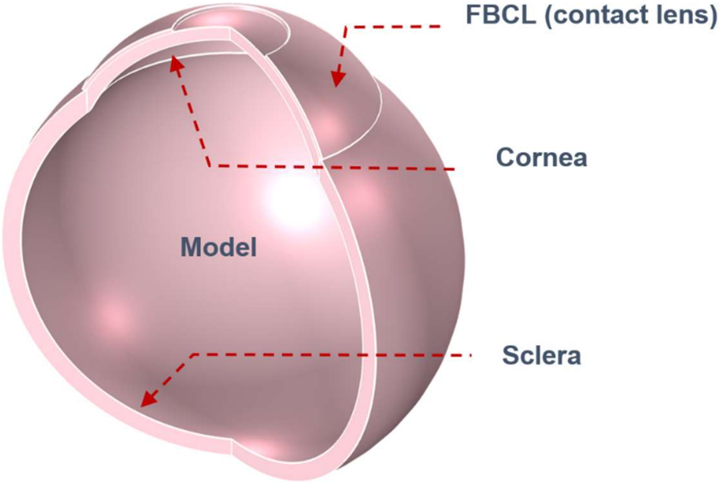

Figure S1. Ocular model incorporating the cornea, sclera, and the FBCL, constructed using COMSOL. The corneal thickness is set at 0.7 mm, scleral thickness at 1 mm, and FBCL thickness at 0.2 mm. During the simulations, the fluid-structure interaction between the aqueous humor and intraocular tissues was neglected, and only the elastic deformation of the tissues was considered. The cornea, sclera, and annular contact lens were all modeled as linear elastic materials, with the Young's modulus and Poisson's ratio set as follows: for the cornea, 0.8 MPa and 0.44, for the sclera, 2.7 MPa and 0.47, and for the annular contact lens, 0.55 MPa and 0.4.

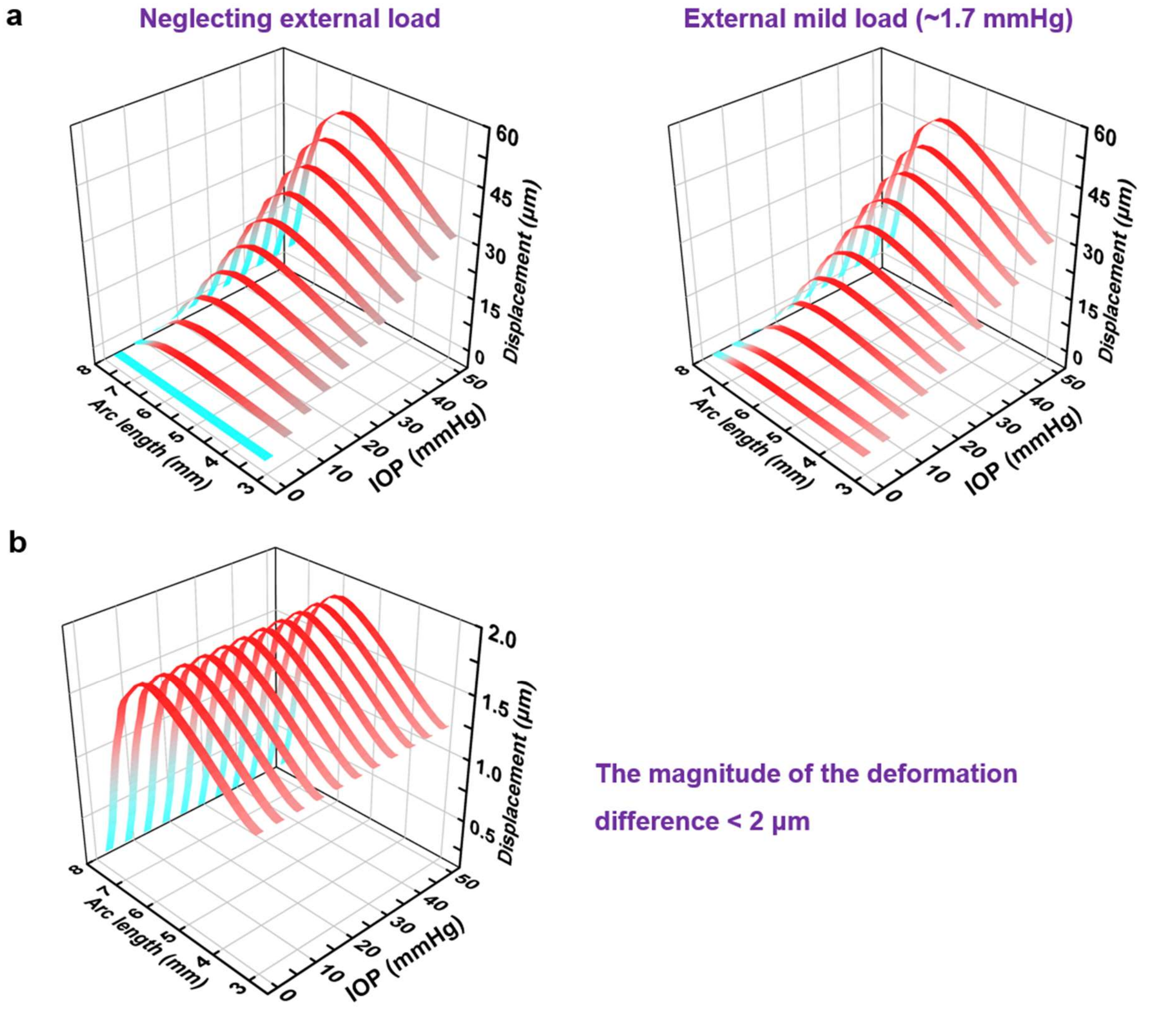

Figure S2. Deformation simulation of the FBCL under mild ocular load. **a)** Deformation simulations of the FBCL under different IOP levels without external load and with mild external load. **b)** Difference in deformation magnitude of the FBCL under the two conditions.

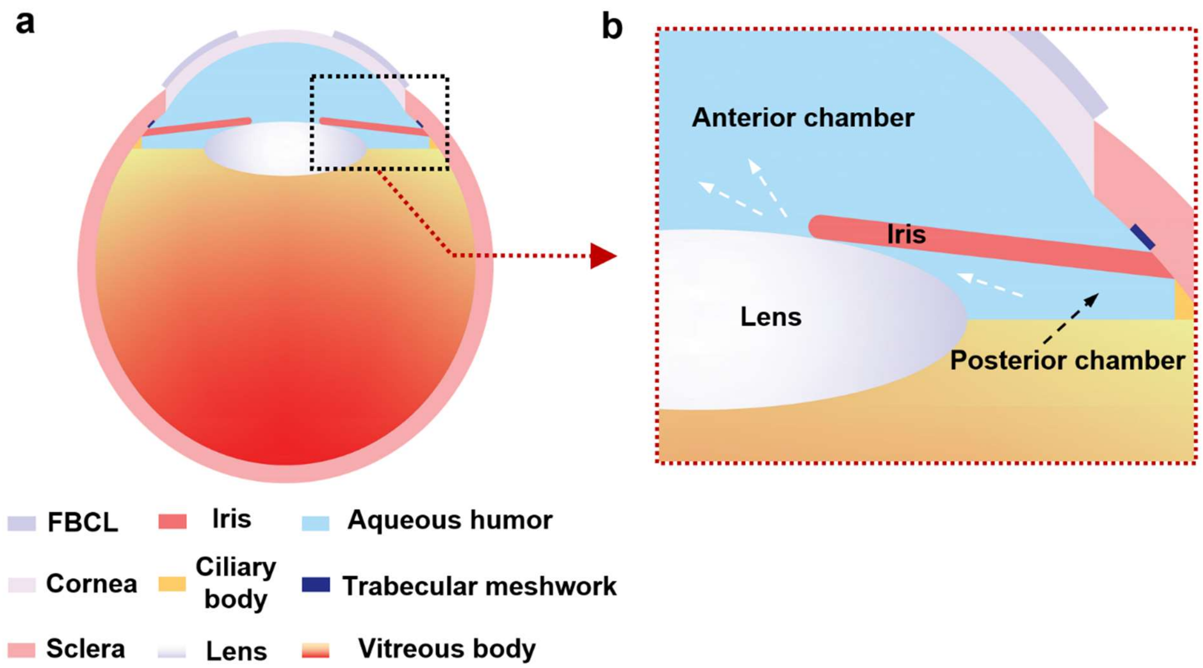

Figure S3. Intraocular drug transport model with the annular FBCL. **a)** Composition of the various parts in the model. **b)** Enlarged detail of the anterior and posterior chambers connected to each other.

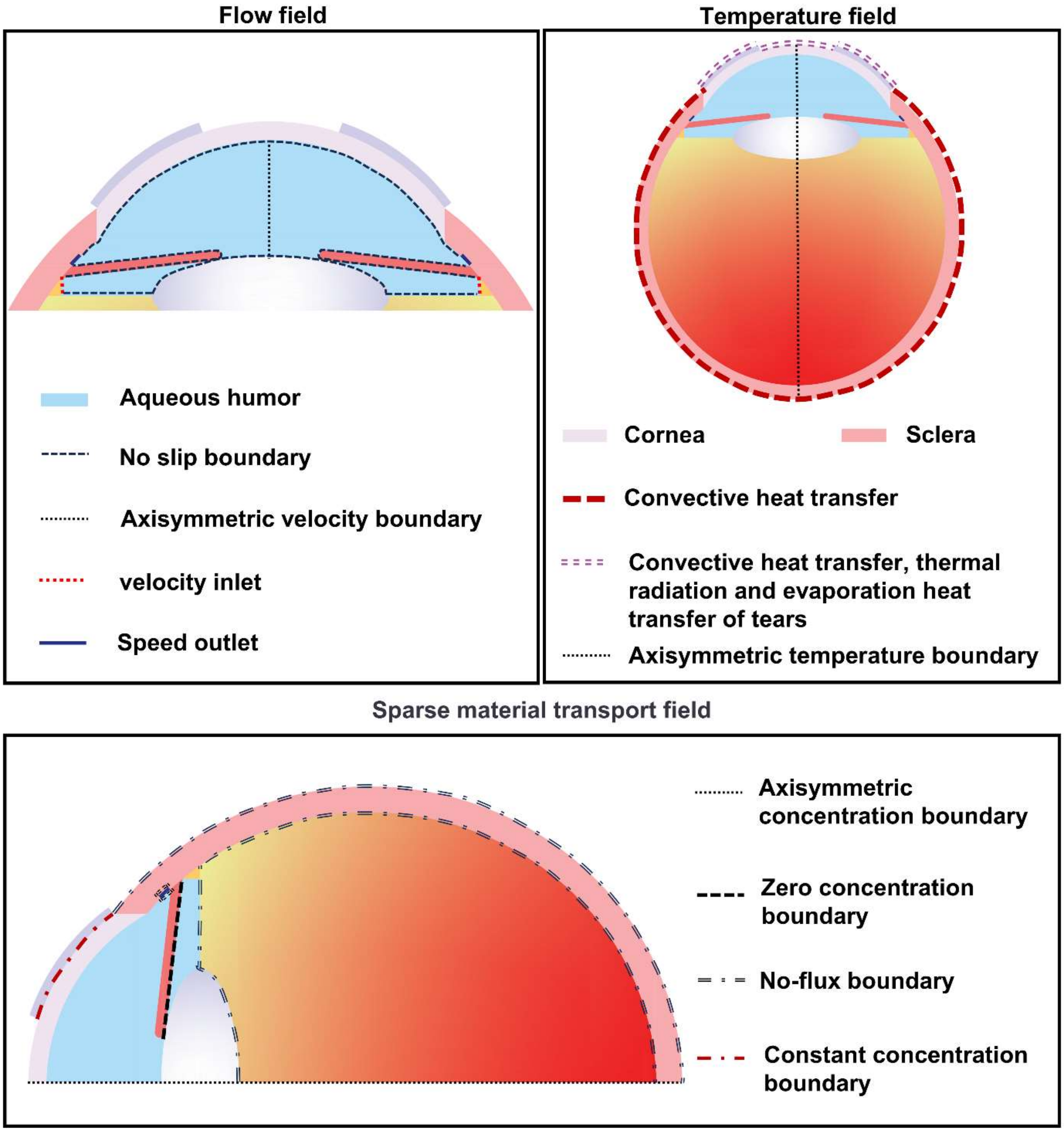

Figure S4. Boundary conditions of the intraocular drug transport model.

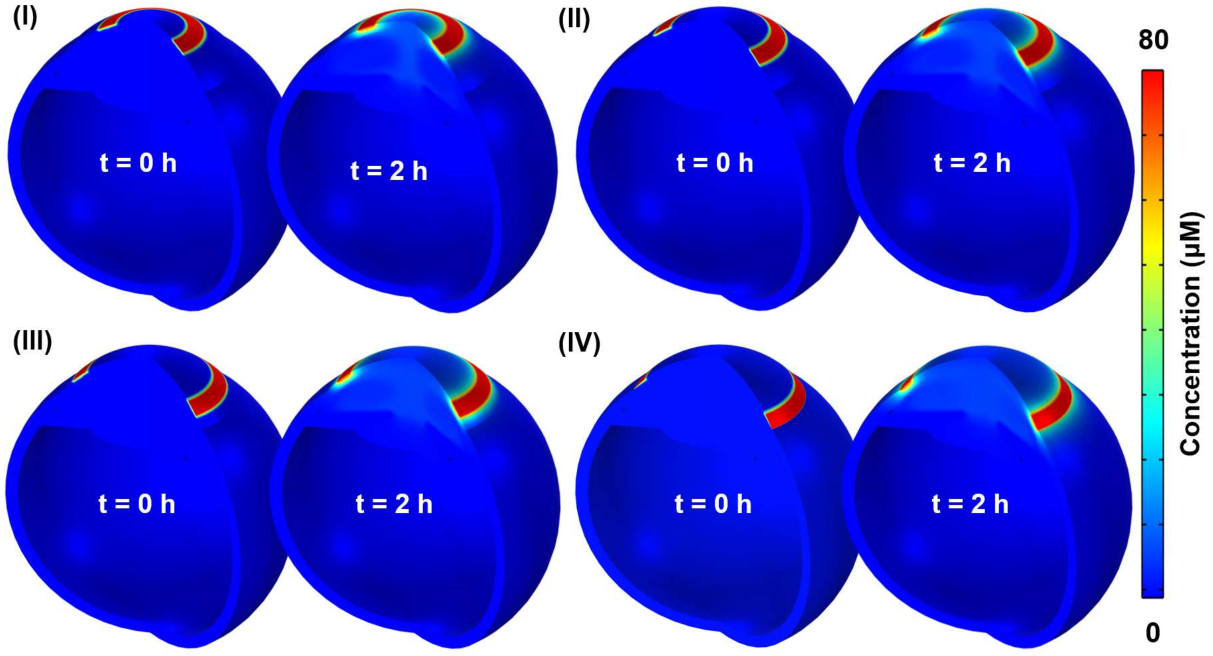

Figure S5. Drug concentration distribution within the ocular region after application at different locations on the cornea.

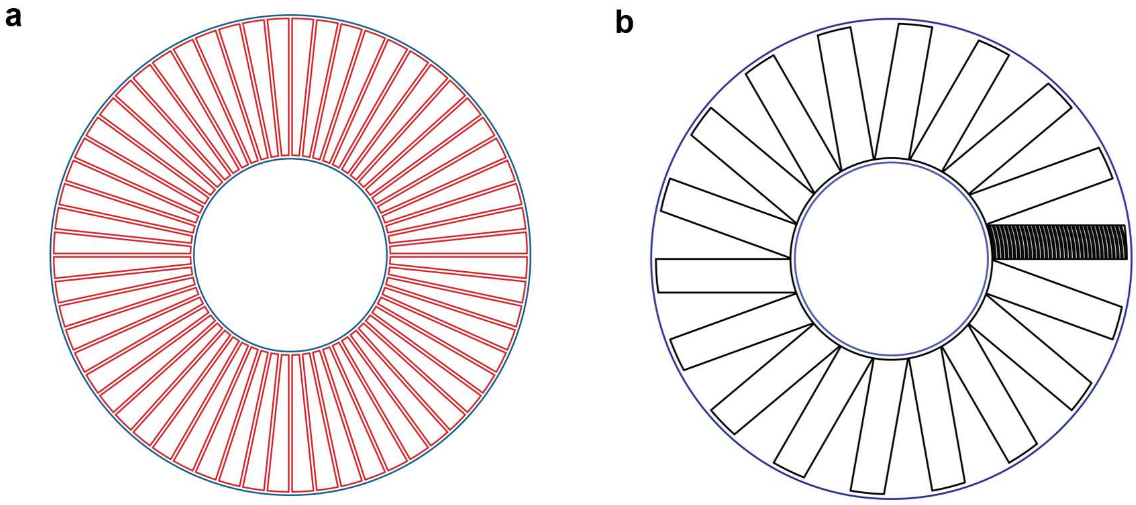

Figure S6. Layout choices in the design process of the FBCL. **a)** Spiderweb-like layout. **b)** Constant-width radial layout.

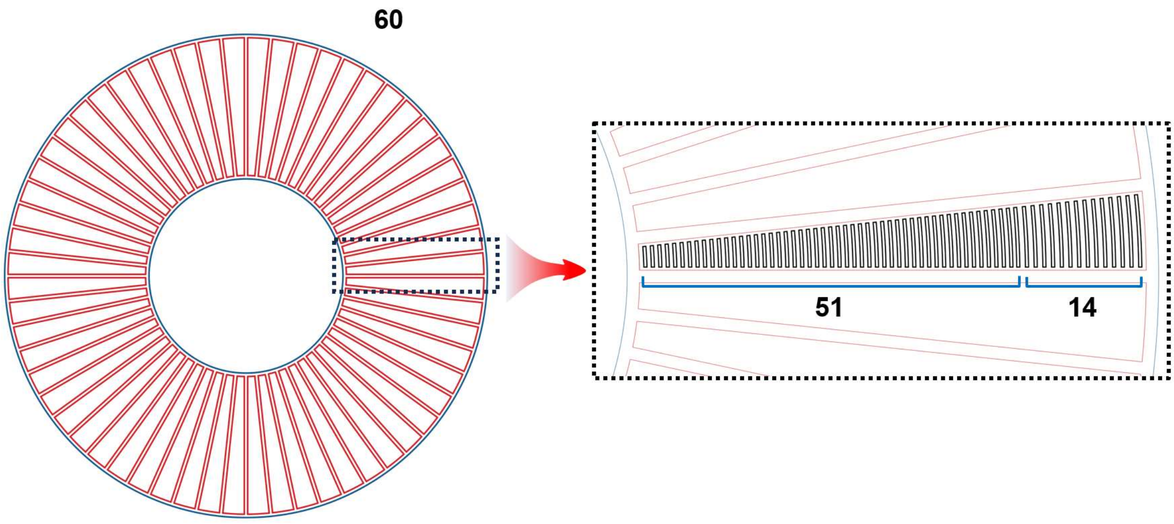

Figure S7. Design parameters of microstructural units in the planar microstructure layout.

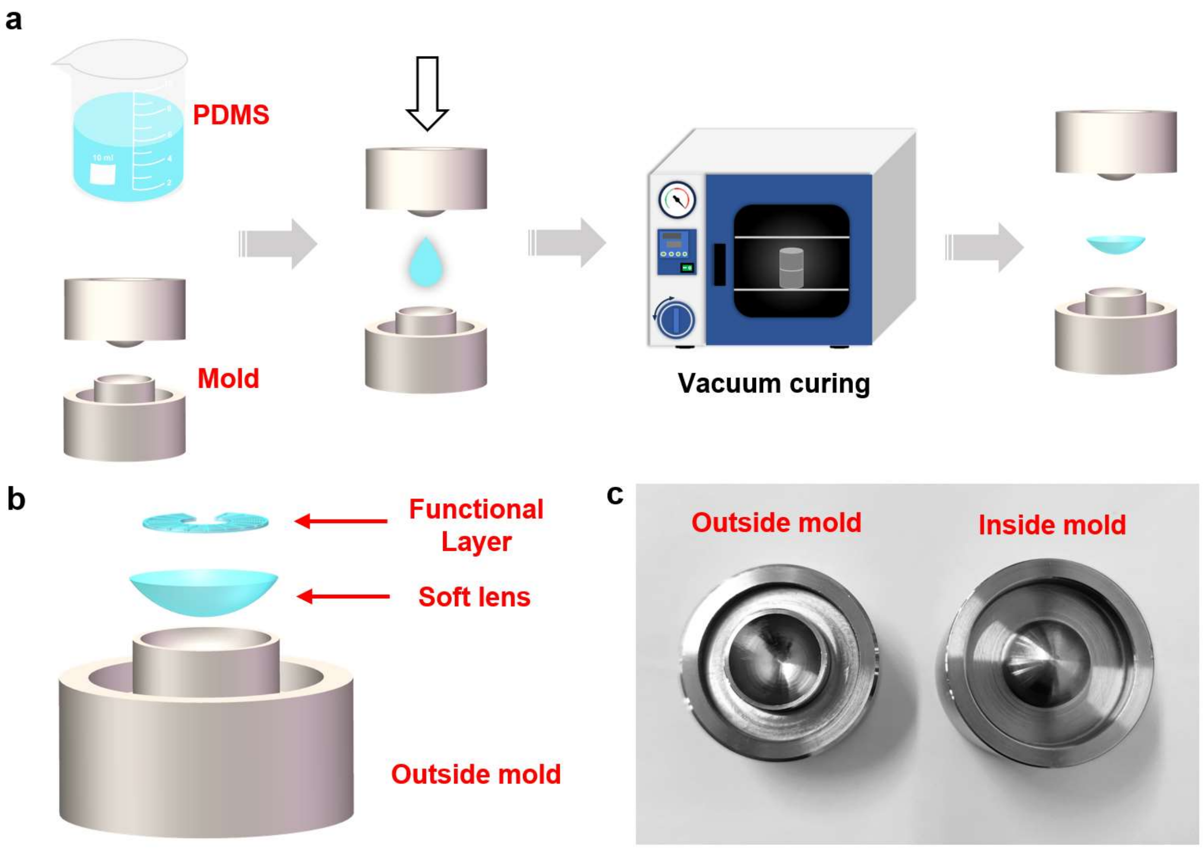

Figure S8. Manufacturing of the contact lens base and its assembly with the PDMS microstructure layer. **a)** Manufacturing contact lens using stainless steel molds. **b)** Assembly of microstructures with soft contact lens. **c)** Physical image of the stainless steel metal mold.

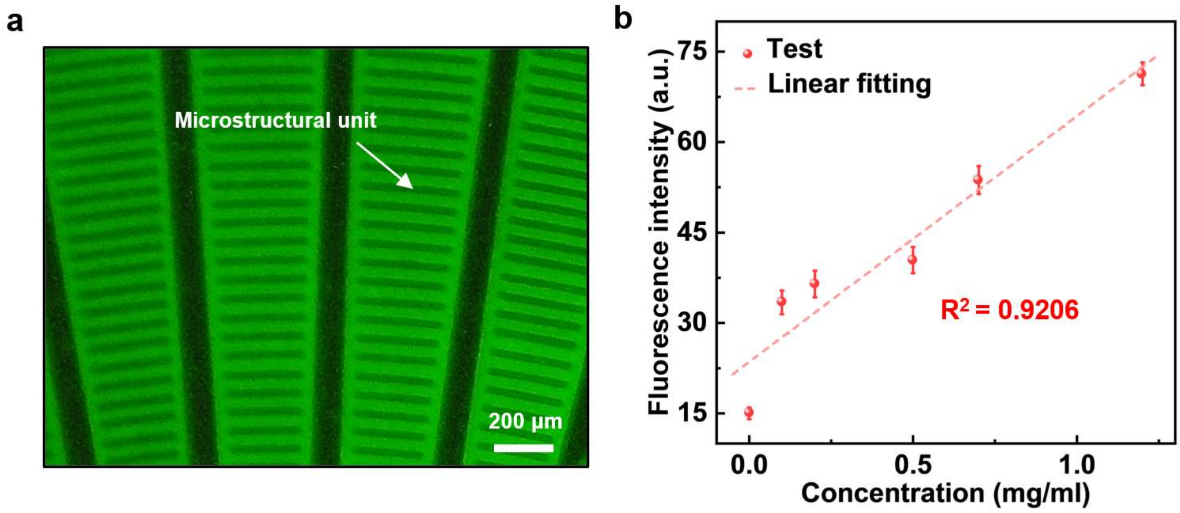

Figure S9. Drug loading image and fluorescence calibration. **a)** Fluorescence image of the microstructure. **b)** Linear fitting of average fluorescence intensity to drug concentration ($n = 3$).

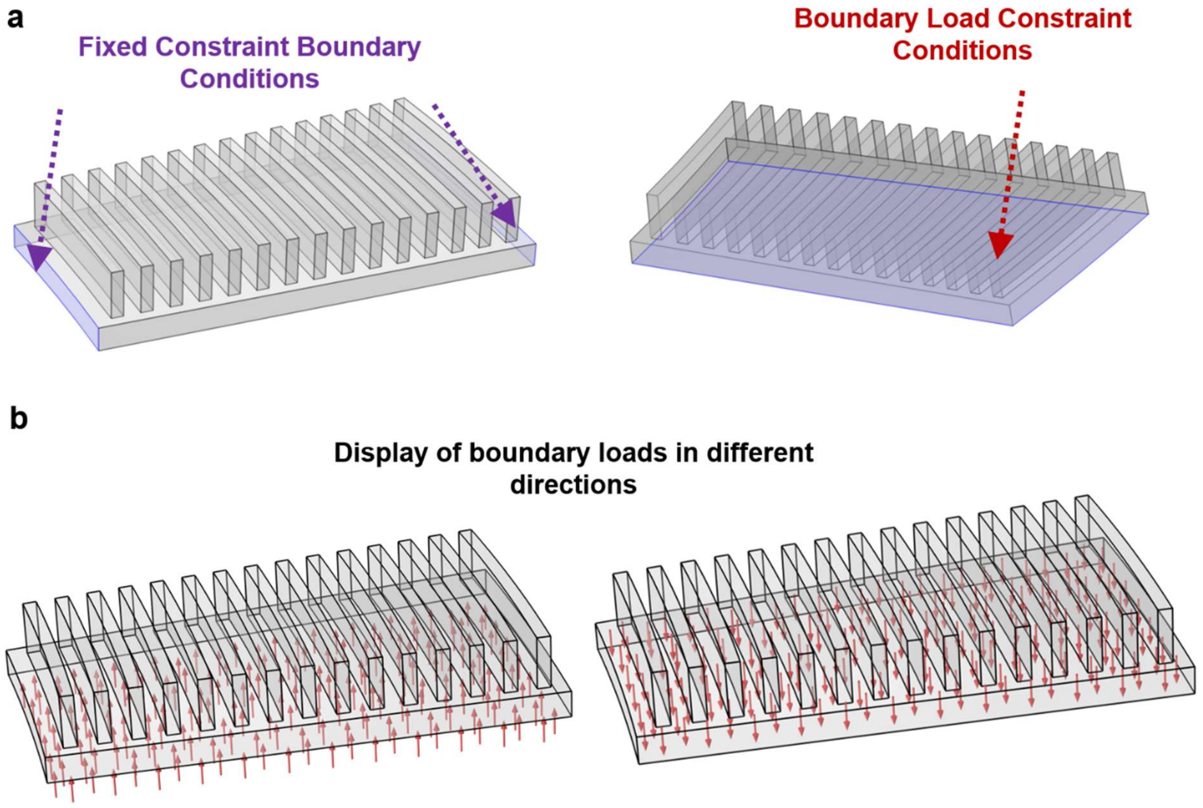

Figure S10. Boundary conditions and application methods of different boundary loads in the microstructure deformation simulation. **a)** Boundary conditions for the microstructure deformation simulation. **b)** Application methods of different boundary loads.

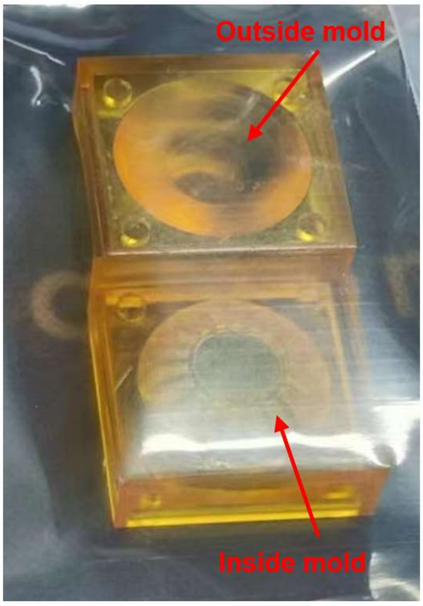

Figure S11. Physical image of the curved mold prepared using 3D printing. The interstices of the microstructure in the mold measure 150 μm.

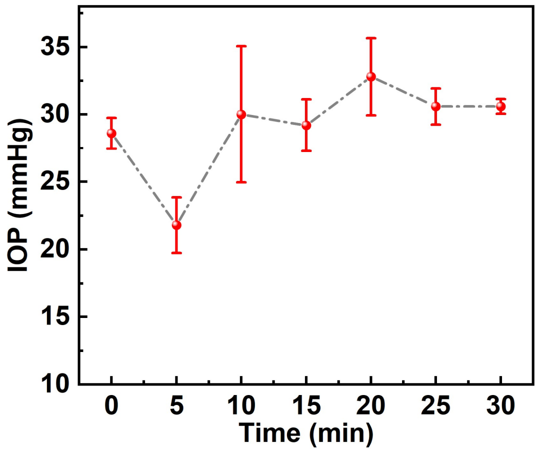

Figure S12. IOP variations in the rabbit eye after high-IOP induction surgery (n = 5).

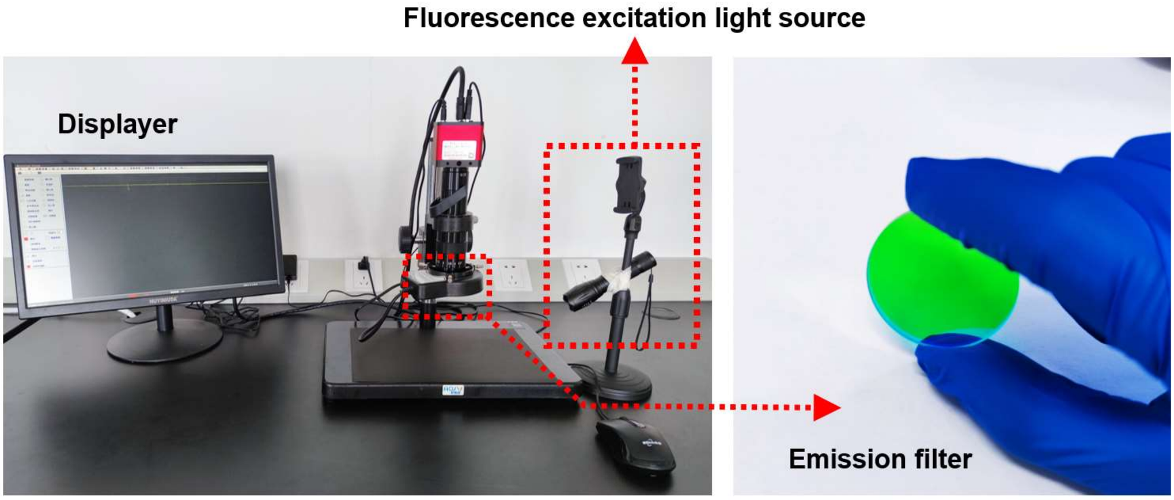

Figure S13. Modified fluorescence microscope experimental platform. The entire experiment is conducted in a dark environment.

Table S1. Comparison between this work and previous related drug delivery devices

Complexity of drug preparing and loading	Cores of drug release principle	Suitability for long-term medication to overcome adherence	Regulate or control drug release	Flexibility in initiating and interrupting drug release	Ref.
High	Nanoparticles	Yes	No	High	[1]
High	Daylight-mediated	Yes	Yes	High	[2]
High	Molecular imprinting	Yes	No	High	[3]
Low	Blinking release	No	Yes	High	[4]
High	Electrically triggered	No	Yes	High	[5]
High	Temperature triggered	Yes	Yes	High	[6]
Middle	Silicon nano-needles	Yes	No	Low	[7]
Middle	Electrically triggered	No	Yes	High	[8]
Low	Magnetic fields	No	Yes	High	[9]
Lowest	Pressure and blinking	Yes	Yes	High	This work

Movie S1: Demonstration of cyclic fluctuations in intraocular pressure of pig eyes under external load.

References

[1] J. Sun, Y. Lei, Z. Dai, X. Liu, T. Huang, J. Wu, Z. P. Xu, X. Sun, *ACS Appl. Mater. Interfaces* **2017**, *9*, 7990.

[2] C. Mu, M. Shi, P. Liu, L. Chen, G. Marriott, *ACS Cent. Sci.* **2018**, *4*, 1677.

[3] Z. Chu, C. Xue, K. Shao, L. Xiang, X. Zhao, C. Chen, J. Pan, D. Lin, *ACS Appl. Bio Mater.* **2021**, *5*, 243.

[4] Z. Du, G. Zhao, A. Wang, W. Sun, S. Mi, *ACS Appl. Polym. Mater.* **2022**, *4*, 7290.

[5] T. Y. Kim, J. W. Mok, S. H. Hong, S. H. Jeong, H. Choi, S. Shin, C.-K. Joo, S. K. Hahn, *Nat. Commun.* **2022**, *13*, 6801.

[6] S.-H. Lee, K.-S. Shin, J.-W. Kim, J.-Y. Kang, J.-K. Kim, *Transl. Vision Sci. Technol.* **2020**, *9*, 1.

[7] W. Park, V. P. Nguyen, Y. Jeon, B. Kim, Y. Li, J. Yi, H. Kim, J. W. Leem, Y. L. Kim, D. R. Kim, *Sci. Adv.* **2022**, *8*, eabn1772.

[8] C. Yang, Q. Wu, J. Liu, J. Mo, X. Li, C. Yang, Z. Liu, J. Yang, L. Jiang, W. Chen, H.-J. Chen, J. Wang, X. Xie, *Nat. Commun.* **2022**, *13*, 2556.

[9] C. Wang, J. Park, *Micro Nano Lett.* **2020**, *8*, 1.
